# Changes in canine C-reactive protein levels following orthopaedic surgery: a prospective study

**DOI:** 10.1186/s13028-019-0468-y

**Published:** 2019-07-01

**Authors:** Nobuo Kanno, Noriyuki Hayakawa, Shuji Suzuki, Yasuji Harada, Takuya Yogo, Yasushi Hara

**Affiliations:** 1 Division of Veterinary Surgery, Department of Veterinary Science, Faculty of Veterinary Medicine, Nippon Veterinary and Life Science University, 1-7-1 Kyonan-cho, Musashino, Tokyo 180-8602 Japan; 2 Veterinary Medical Teaching Hospital, Nippon Veterinary and Life Science University, 1-7-1 Kyonan-cho, Musashino, Tokyo 180-8602 Japan

**Keywords:** Canine inflammatory biomarker, Clinical study, Postoperative complications

## Abstract

C-reactive protein (CRP) is a powerful biomarker for inflammation, infection and sepsis. However, no reports have investigated canine CRP (c-CRP) concentration changes after orthopaedic procedures. If c-CRP changes were better characterized, it may be possible to identify postoperative complications more quickly. The purpose of this study was to clarify the characteristic changes in serum c-CRP after orthopaedic surgery in dogs. Blood samples were collected from 98 dogs on Day 0 (preoperatively), and then on Days 1, 4, 7, 10 and 13 postoperatively. Day 1 blood sampling was performed 12–24 h postoperatively. We classified the dogs into four groups based on changes in c-CRP pre- to postoperatively. Group 1 dogs showed a peak c-CRP concentration on Day 1, followed by decreases of ≥ 1 mg/dL. Group 2 dogs showed changes in c-CRP concentration by Day 4 that were within ± 1 mg/dL compared with Day 1. Dogs in Group 3 showed a peak c-CRP concentration on Day 4, followed by decreases of ≥ 1 mg/dL. Group 4 dogs showed an initial decrease in c-CRP, then an increase of ≥ 1 mg/dL. Group 1 was the largest group, with 63 dogs. c-CRP on Days 0, 1, 4, 7, 10 and 13 was 0.83 ± 1.03 mg/dL, 8.10 ± 3.15 mg/dL, 3.76 ± 1.94 mg/dL, 1.63 ± 0.92 mg/dL, 0.96 ± 0.70 mg/dL and 0.68 ± 0.51 mg/dL, respectively. Serum c-CRP concentration on Day 1 was significantly higher than at every other timepoint (P < 0.001). In Group 2, surgical site complications were confirmed in 9/15 dogs. In Group 3, surgical site complications were confirmed in 7/14 dogs. In Group 4, two surgical site problems and three surgical site infections were observed, and visceral disease was found in one dog. In Group 1, peak c-CRP was seen on Day 1 postoperatively in 63 dogs (64%), with c-CRP level decreasing by half at each subsequent measurement, which may describe a typical c-CRP change in orthopaedic patients. If deviation from this typical change is observed postoperatively, as in Groups 2–4, this may suggest possible complications.

## Findings

In small animal orthopaedic surgery, it is important to diagnose postoperative complications early. Overall, surgical site infection rates in small animals range from 2.83 to 5.8% [1–3]. C-reactive protein (CRP) is a powerful biomarker for inflammation, infection and sepsis, and is widely used in both human and veterinary medicine [4]. Clinical serum canine CRP (c-CRP) measurement has been performed for over 30 years [5], and increased serum c-CRP concentrations are known to occur with several types of infection [6–8].

Concentrations of c-CRP begin to increase less than 6 h postoperatively, and maximum concentrations have been observed approximately 12–24 h postoperatively [9–11]. If normal changes in c-CRP after orthopaedic surgery were better characterized, it may be possible to identify postoperative complications more quickly.

The purpose of the present study was to characterize the changes in c-CRP after orthopaedic surgery in dogs. We expected to see maximum c-CRP concentrations in the first 12–24 h after surgery, followed by a gradual decrease.

This was a prospective study. Data were collected from 98 dogs treated for orthopaedic disorders at our institution between February 2014 and September 2016. The dog breeds varied, with a predominance of Toy Poodles (n = 30). Other breeds included Labrador Retriever (n = 8), mixed breed (n = 8), Miniature Dachshund (n = 6), Bernese Mountain Dog (n = 5), Pomeranian (n = 5), Chihuahua (n = 4), Border Collie (n = 3), Golden Retriever (n = 3), Italian Greyhound (n = 3), Jack Russell Terrier (n = 3), Shiba Inu (n = 3), Beagle (n = 2), Siberian Husky (n = 2), Welsh Corgi (n = 2), Yorkshire Terrier (n = 2) and English Bulldog, Cavalier King Charles Spaniel, French Bulldog, German Shorthaired Pointer, Great Dane, Maltese, Shetland Sheepdog, St. Bernard and White Shepherd (n = 1 each).

Blood samples were collected on Day 0 (before surgery), then on Days 1, 4, 7, 10 and 13 postoperatively. Day 1 blood sampling was performed 12–24 h postoperatively. Blood samples were collected slowly from the jugular vein or lateral saphenous vein into serum separating tubes (Insepack SMD760CG; Sekisui Medical, Tokyo, Japan). Serum samples were then centrifuged at 2000*g* for 8 min (Tabletop centrifuge-4200; Kubota, Japan). c-CRP levels were measured using a latex agglutination immunoassay method with an automatic analyser (Hitachi Automatic Analyser 7180; Hitachi, Tokyo, Japan). The reagent used in the present study was the Canine CRP NEO (canine C-Reactive Protein Measurement Kit; Shima Laboratories Co., Ltd., Tokyo, Japan). The assay range of this kit is 0.2–20 mg/dL [12]; the reference value for c-CRP in our institution is ≤ 1.0 mg/dL.

All dogs were cared for according to the perioperative care protocol of the Veterinary Medical Teaching Hospital, Nippon Veterinary and Life Science University for postorthopaedic patients (Table 1). Bandages were applied for 1 week but were applied longer when the surgeon determined it was necessary. Radiographic evaluations were performed immediately after surgery, and on Day 7 and Day 14. If the patient was discharged earlier than 14 days after surgery, the radiographic evaluations were performed on the day of discharge. After at least 10 days postoperatively, and once the surgical incision was healed, we removed the sutures 1 day before or on the day of discharge from hospital.

**Table 1:** Basic perioperative care protocol for postorthopaedic patients in our institution

Day	0	1	2	3	4	5	6	7	8	9	10	11	12	13	14
TPR^a^	●	●	●	●	●	●	●	●	●	●	●	●	●	●	●
Bandage change^b^	●			●			●	●							
Radiography	●							●							●
c-CRP measurement	●	●			●			●			●			●	
Suture removal															●

The day each intervention was performed is indicated by a black dot (●)

*TPR* temperature, pulse and respiratory rate, *c-CRP* canine C-reactive protein

^a^TPR was measured twice each day

^b^If necessary, the bandage was changed twice each day

This research was approved by the ethics committee of the Veterinary Medical Teaching Hospital, Nippon Veterinary and Life Science University, Japan.

We classified the dogs into one of four groups based on changes in c-CRP. Group 1 dogs showed a peak c-CRP on Day 1, and then levels continued to decrease during each subsequent measurement period. Group 2 dogs showed changes in c-CRP concentration by Day 4 that were within ± 1 mg/dL compared with Day 1. Group 3 dogs showed a peak c-CRP on Day 4 followed by decreasing levels during each subsequent measurement period. Group 4 dogs showed a peak c-CRP on Day 1 or Day 4, which decreased then increased again.

Statistical analysis was performed between measurement days within Groups 1, 2 and 3 and between Groups 1, 2 and 3 for each measurement day. The normality of each c-CRP concentration in Group 1, 2 and 3 was confirmed using the Shapiro–Wilk’s test. The homoscedasticity of each value was then examined using Levene’s test. Homoscedasticity was confirmed for each value, then significant differences were confirmed using Tukey’s honestly significant difference test. When homoscedasticity was not observed for a value, significant differences were confirmed using the Games–Howell test. Data were expressed as the mean ± standard deviation, and P < 0.05 was considered statistically significant. The Statistical Package for the Social Sciences version 16.0.1 (SPSS, IBM Inc., Armonk, NY, USA) was used to perform all statistical analyses.

The pre- and postoperative c-CRP concentrations, number of dogs, and the surgical procedures in Groups 1, 2 and 3 are shown in Table 2. Table 2 also includes the events that may have affected the c-CRP concentrations. Group 4 dogs’ characteristics, surgical procedures, postsurgical findings and the pre- and postoperative c-CRP concentrations are summarized in Table 3.

**Table 2:** Pre- and postoperative serum c-CRP concentration, number of dogs, surgical procedures and postsurgical events in Group 1, 2 and 3

	Day 0	Day 1	Day 4	Day 7	Day 10	Day 13
Group 1						
Type	Peak c-CRP concentration on Day 1 and then continued to decrease					
Mean ± SD	0.83 ± 1.03^b,c,e^	8.10 ± 3.15^a,c,e,g,i^	3.76 ± 1.94^a,b,e,g,i^	1.63 ± 0.92^a,b,c,g,i^	0.96 ± 0.70^b,c,e^	0.68 ± 0.51^b,c,e^
n	63	63	63	63	63	45
Surgical procedures	Corrective osteotomy (n = 24), reduction of dislocation (n = 13), internal fracture fixation (n = 11), non-union repair (n = 6), ostectomy (n = 5), arthrodesis (n = 3), joint replacement (n = 1)					
Event	Non					
Group 2						
Type	Peak c-CRP concentration on Day 1and Day 4, and then continued to decrease					
Mean ± SD	0.84 ± 1.10^b,c,e^	5.88 ± 2.27^a,e,g,i,x^	5.99 ± 2.21^a,e,g,i,y^	2.18 ± 1.08^a,b,c,i^	1.09 ± 0.96^b,c^	0.77 ± 0.55^b,c,e^
n	15	15	15	15	15	11
Surgical procedures	Corrective osteotomy (n = 9), reduction of dislocation (n = 2), non-union repair (n = 1), ostectomy (n = 1), arthrodesis (n = 2)					
Event	Swelling and/or heat at the surgical site (n = 9), visceral disease (n = 3), no identifiable causes (n = 3)					
Group 3						
Type	Peak c-CRP concentration on Day 4 and then continued to decrease					
Mean ± SD	0.49 ± 0.39^b,c,e,g^	6.56 ± 3.69^a,g,i^	9.68 ± 5.67^a,f,g,i,y^	3.76 ± 1.75^a,d,h,i,y,z^	1.67 ± 0.85^a,b,c,f^	1.43 ± 0.96^b,c,e^
n	14	14	14	14	14	11
Surgical procedures	Corrective osteotomy (n = 7), reduction of dislocation (n = 2), non-union repair (n = 1), ostectomy (n = 2), arthrodesis (n = 2)					
Event	Swelling and/or heat sensation at the surgical site (n = 5), visceral disease (n = 4), surgical site problems (n = 2), non-surgical site problems (n = 2), no identifiable causes (n = 2)					

The reported values are the mean ± standard deviation (SD). c-CRP values are in mg/dL

*c-CRP* canine C-reactive protein

^a^Games–Howell test; P < 0.01 indicates a significant difference vs. Day 0

^b^Games–Howell test; P < 0.01 indicates a significant difference vs. Day 1

^c^Games–Howell test; P < 0.01 indicates a significant difference vs. Day 4

^d^Games–Howell test; P < 0.05 indicates a significant difference vs. Day 4

^e^Games–Howell test; P < 0.01 indicates a significant difference vs. Day 7

^f^Games–Howell test; P < 0.05 indicates a significant difference vs. Day 7

^g^Games–Howell test; P < 0.01 indicates a significant difference vs. Day 10

^h^Games–Howell test; P < 0.05 indicates a significant difference vs. Day 10

^i^Games–Howell test; P < 0.01 indicates a significant difference vs. Day 13

^x^Tukey’s honestly significant difference test; P < 0.05 indicates a significant difference vs. Group 1

^y^Games–Howell test; P < 0.01 indicates a significant difference vs. Group 1

^z^Games–Howell test; P < 0.05 indicates a significant difference vs. Group 2

**Table 3:** Group 4 dogs’ characteristics, surgical procedures, postsurgical findings and pre- and postoperative canine C-reactive protein concentrations

Case number	Case information				Concentrations of serum c-CRP (mg/dL)					
	Breed	Sex	Age (month)	BW (kg)	Day 0	Day 1	Day 4	Day 7	Day 10	Day 13
2	Miniature Dachshund	Female	86	6.0	1.6	6.0	2.6	0.3	0.3	2.5
Surgical procedure	Internal fixation for closed femoral fracture									
Specific findings	A bacterial infection was confirmed from femoral abrasions									
5	Toy Poodle	Female	23	1.5	1.0	5.4	3.8	1.5	4.1	3.2
Surgical procedure	Internal fixation for radioulnar non-union									
Specific findings	A bacterial infection was confirmed from specimens collected intraoperatively									
10	Toy Poodle	Female	50	2.8	0.3	5.9	8.5	4.7	1.4	4.2
Surgical procedure	Internal fixation for radioulnar non-union, bipedicle flap									
Specific findings	Partial necrosis of the bipedicle flap was observed 13 days after surgery									
13	Bulldog	Female	81	24.3	0.3	11.8	3.7	1.3	2.9	5.1
Surgical procedure	Tibial plateau leveling osteotomy									
Specific findings	Exudate was observed from the surgical sutures 12 days after surgery, although no bacteria were cultured from the exudate									
59	Pomeranian	Female	47	2.8	1.7	9.8	4.0	2.5	2.6	4.3
Surgical procedure	Internal fixation for radioulnar non-union									
Specific findings	Bacterial infection was confirmed from fluid within the surgical field									
64	Toy Poodle	Spayed	40	4.1	1.2	15.9	12.0	7.8	9.9	3.2
Surgical procedure	Femoral corrective osteotomy for lateral patellar luxation									
Specific findings	Anorexia and melaena were observed 5 days postoperatively									

*c-CRP* canine C-reactive protein, *BW* body weight

In Group 1, c-CRP concentration on Day 1 was significantly higher than at all other timepoints (P < 0.001). Additionally, c-CRP concentrations on Day 10 and Day 13 were not significantly different from those on Day 0 (Table 2). There were no confirmed events that could have affected the postoperative c-CRP concentrations. However, two dogs had confirmed bacterial infection based on the culture and sensitivity of bone fragments collected during surgery, and both dogs underwent surgical repair of a nonunion. In Group 2, 12/15 dogs experienced events that may have affected the c-CRP concentrations (Table 2). Diarrhoea, vomiting and haematuria were confirmed as signs of visceral disease in these 12 dogs. No dogs in Group 2 were suspected of having surgical site infections. In Group 3, 12/14 dogs experienced events that may have affected the c-CRP concentrations (Table 2). Gastric dilation was confirmed in addition to diarrhoea, vomiting and haematuria, which were signs of visceral disease. Surgical site problems included self-injury and partial necrosis of the suture line of the bipedicle flap that was used to repair radioulnar nonunion. Additionally, inflammation of the intravenous catheter site was identified as a nonsurgical site problem. Swelling and heat at the surgical site and visceral disease both occurred in one dog.

Group 4 included six dogs, numbered 2, 5, 10, 13, 59 and 64 (Table 3). For all dogs except dog 64, antibiotics were based on drug susceptibility tests or clinical symptoms. A decline in c-CRP concentration was observed after treatment in all dogs.

Sixty-three dogs (64%) showed a peak c-CRP concentration on Day 1 after surgery, consistent with previous studies of patients undergoing soft tissue surgery or spinal surgery [9–11]. Additionally, the c-CRP concentration in Group 1 decreased to preoperative levels within 10 days postoperatively. Studies evaluating postoperative c-CRP levels in orthopaedic surgery have shown a peak at 24 h postoperatively, and a marked decrease by Day 8 after surgery [13, 14]. c-CRP concentrations 7 days after surgery were significantly higher than preoperative levels, in our study. The postoperative c-CRP concentration decreased by approximately half at every measurement after Day 1, in our study, which may define typical c-CRP changes in orthopaedic patients.

A total of 35.7% (35/98) of the dogs did not belong to Group 1, and 86% (30/35) of these dogs had problems associated with changes in c-CRP. A previous study measured c-CRP in dogs undergoing ovariohysterectomy for pyometra [15]. This previous study showed that dogs with infections and postoperative complications showed significantly higher postoperative c-CRP concentrations compared with dogs without such complications [16]. Five dogs in our study had confirmed bacterial infection based on specimens collected during surgery; two of these dogs were classified into Group 1, while three dogs were classified into Group 4. One of the two dogs classified into Group 1 had tibial nonunion, and methicillin-resistant *Staphylococcus aureus* was isolated from bone chips collected during surgery. In this dog, 25 mg/kg cefmetazole sodium (CMZ) was administered twice a day intravenously intra- and postoperatively, based on culture and sensitivity. The other dog, which had a radioulnar nonunion, received 25 mg/kg CMZ twice daily intravenously and 5 mg/kg enrofloxacin once daily subcutaneously. *Pseudomonas aeruginosa* was isolated from a bone chip collected during surgery, and the antibiotic was changed to ceftazidime hydrate 25 mg/kg three times a day intravenously over 1 h; however, the antibiotic sensitivity testing did not include CMZ and enrofloxacin, so it is unclear whether these antibiotic agents were effective for *P. aeruginosa*. Postoperative c-CRP levels in the two dogs in Group 1 may have decreased over time because of the dogs’ response to the intra- and postoperative antibiotics.

Changes in postoperative c-CRP are likely to be affected by the presence of surgical site infections; however, it was not possible to evaluate the influence of surgical site infection on postoperative c-CRP changes because of the small number of dogs, in our study. However, a recent study that measured c-CRP after tibial plateau levelling osteotomy reported that c-CRP levels in dogs with clinical signs of postoperative infection deviate from expected levels on postoperative Day 6 [17].

Dogs with elevated c-CRP with concurrent marked swelling and/or heat at the surgical site were observed in Groups 2 and 3 in 60.0% and 35.7% of dogs, respectively, and visceral disease was observed in 20.0% and 28.6% of dogs, respectively. This suggests that if the Day 4 c-CRP concentration does not decrease compared with that on Day 1, clinicians should consider the presence of visceral disease, as well as contamination of the surgical site. In fact, c-CRP concentration on Day 4 in Group 1 was significantly lower compared with Group 2 and 3, in this study. In other words, we may be able to classify dogs regarding the presence or absence of complications by observing the pattern of c-CRP change between Day 0–1, and between Day 1–4, postoperatively. However, our results also indicate that it is difficult to identify the type of complication from the pattern of c-CRP change.

There are certain limitations in the present study. First, 22 dogs had preoperative c-CRP concentrations higher than the reference value, namely 14, 4, 1 and 3 dogs in Group 1, 2, 3 and 4, respectively. Events with a possible effect on preoperative c-CRP levels included five bone fractures, four infections and one revision surgery. However, in the other 12 dogs, the cause of the higher preoperative c-CRP concentration could not be identified. Second, we were not able to clarify how preoperative inflammation affects changes in c-CRP concentration after surgery. Third, we did not evaluate liver function, which is the organ that produces c-CRP. Fourth, we focused only on c-CRP concentration as an inflammatory marker, in this study; therefore, white blood cell counts, fibrinogen, serum amyloid A protein and α_1_-acid glycoprotein, which are known to increase with inflammation, were not included in our evaluations, and it is unclear if these inflammatory markers change similarly to c-CRP concentration after orthopaedic surgery in dogs. Fifth, anti-inflammatory drugs such as nonsteroidal anti-inflammatory drugs and steroids were not permitted within the measurement period. Therefore, the number of dogs in our study was relatively small, and it was impossible to compare all procedures. Additionally, the effect on c-CRP of nonsteroidal anti-inflammatory drugs, which are often used in orthopaedic patients, could not be determined. However, one report suggests that carprofen or meloxicam administration in dogs undergoing elective ovariohysterectomy does not affect postoperative c-CRP concentrations [18].

The present study showed a characteristic pattern of c-CRP changes after orthopaedic surgery. If deviation from this characteristic change is observed, clinicians should suspect the possibility of complications such as surgical site infections, and further assess the patient.

## Data Availability

The datasets used and/or analysed during the current study are available from the corresponding author on reasonable request.

## Abbreviations

c-CRP: canine C-reactive protein
CMZ: cefmetazole sodium
